# The Antitumor and Immunomodulatory Effect of Yanghe Decoction in Breast Cancer Is Related to the Modulation of the JAK/STAT Signaling Pathway

**DOI:** 10.1155/2018/8460526

**Published:** 2018-11-18

**Authors:** Dan Mao, Lei Feng, Hui Gong

**Affiliations:** ^1^Department of Integrated Traditional Chinese & Western Medicine, The Second Xiangya Hospital, Central South University, Changsha, Hunan 410000, China; ^2^Department of Oncology, Liuyang Hospital of Traditional Chinese Medicine, Liuyang, Hunan 410300, China; ^3^Department of Oncology, Hunan Academy of Traditional Chinese Medicine Affiliated Hospital, Changsha, Hunan 410006, China

## Abstract

**Background:**

Yanghe decoction (YHD) has been used in the treatment of breast cancer for hundreds of years in Asia. However, the underlying mechanisms are currently unknown. The present study aims to evaluate the efficacy of YHD on antitumor and immune system enhancement in a 4T1 mouse breast cancer model and to clarify the antitumor mechanisms of YHD in breast cancer.

**Materials and Methods:**

The YHD was orally administrated for 2 weeks after inoculation. Tumor tissues were then removed, weighed, and homogenized. Flow cytometry was used to detect the number of Myeloid-Derived Suppressor Cells (MDSCs), Natural Killer T Cells (NKTs), and T cell subsets. Quantitative real-time PCR was used to detect the expression of inducible nitric oxide synthase (iNOS) and arginase-1 (ARG-1). Western blot was used to detect the protein expression of signal transducers and the activator of transcription 1 (STAT1), phosphorylated-signal transducers and the activator of transcription 1 (p-STAT1), signal transducers and the activator of transcription 3 (STAT3), and phosphorylated-signal transducers and the activator of transcription 3 (p-STAT3). The expression levels of interleukin-6 (IL-6), transforming growth factor-*β* (TGF-*β*), and interferon-*γ* (IFN-*γ*) were detected using an enzyme linked immunosorbent assay.

**Results:**

We found that the tumor weight of YHD high-dose group was significantly lower compared with the control group (*p*<0.05). The YHD depressed the expression of MDSCs, iNOS, ARG-1, IL-6, TGF-*β*, and p-STAT3 and significantly increased the expression of IFN-*γ*, NKTs, CD4^+^ T cells, and p-STAT1.

**Conclusion:**

Our results showed that The mechanisms of YHD inhibit 4T1 breast tumor growth may be related to downregulating the expression of iNOS and ARG-1, negatively regulating the Janus kinase/STAT3 (JAK/STAT3) pathway by repressing the expression of IL-6 and TGF-*β*. Meanwhile, YHD enhances the immune capacity via increasing the expression of NKTs, CD4^+^ T cells, IFN-*γ*, and p-STAT1.

## 1. Introduction

Breast cancer is one of the most common cancers in females. As social aging intensifies globally, the rate of incidence of breast cancer is also increasing [1]. Over the past decades, comprehensive therapy including surgical operation, chemotherapy, radiotherapy, endocrinotherapy, and targeted therapy has significantly reduced mortality rates. However, most patients have to undergo severe side effects, such as serious destruction of the immune system caused by chemotherapy, which can seriously lower the quality of life. So it is highly necessary to seek effective novel treatments with low adverse reactions and adjuvant therapy.

Traditional Chinese Medicine (TCM) has a remarkable effect in adjuvant therapy for cancer, especially in improving clinical symptoms, prolonging the survival of patients and regulating immune functions [2–4]. As an effective way in preventing and treating cancer [5, 6], TCM has been increasingly used in the past few decades around the world. A large body of research shows that TCM plays an increasingly important role in the whole process of anticancer therapy, which can promote the recovery of patients after surgery, reduce toxic and side effects, or enhance the therapeutic effect of chemotherapy and improve quality of life [7–9].

As a traditional Chinese antitumor medication, Yanghe decoction (YHD) was initially recorded in the ‘Wai Ke Zheng Zhi Quan Sheng Ji' written by Hongxu Wang during the Qing dynasty. It is composed of a preparation of rehmannia root (Shu Di Huang), deerhorn glue (Lu Jiao Jiao), Cinnamomum cassia (Rou Gui), semen brassicae (Bai Jie Zi), ephedra (Ma Huang), charcoal of ginger (Jiang Tan), and radix glycyrrhizae (Sheng Gan Cao). Based on the theory of TCM, the primary pathogenesis of breast cancer is a “deficiency of yang”. YHD can warm yang and dispel cold, therefore, physicians of ancient China treated Ruyan (breast cancer) with YHD for hundreds of years, and it is very effective in improving symptoms and prolonging the survival of patients. Also proven by modern medicine, YHD has an effect on breast cancer and its precancerous lesions [10]. However, the underlying mechanism remains unknown.

Metastasis, invasion, and development of a tumor are related to the tumor microenvironment, which is composed of tumor cells, immune cells, extracellular matrix, and interstitial tissue [11]. There are two core characteristics of the tumor microenvironment: chronic inflammation and immune suppression. Myeloid-Derived Suppressor Cells (MDSCs) are the crucial link between these two factors. As a type of immunosuppressive cells, MDSCs can promote T cell apoptosis and reduce the antitumor activity of natural killer cells. Studies have proven that the immunosuppressive action of MDSCs is related to the expression of some cytokines such as interleukin-6 (IL-6), transforming growth factor-*β* (TGF-*β*), interferon-*γ* (IFN-*γ*), inducible nitric oxide synthase (iNOS), and arginase-1 (ARG-1). These cytokines have been implicated in the activation of Janus kinase/signal transducers and the activators of the transcription (JAK/STAT) signaling pathway. Also, MDSCs produce other cytokines, such as metalloproteinases (MMPs) and vascular endothelial growth factors (VEGF), which can promote angiogenesis and tumor invasion [12–14]. Immune cells play multiple roles in antitumor immunity, with MDSCs promoting tumor progression and immune escape by silencing the immune response. TCM could reverse these immunosuppressions and enhance antitumor immune responses [15–18].

In this study, we treated a 4T1 mouse breast cancer model with YHD to observe the antitumor effects on the immune system. The related mechanisms are also discussed.

## 2. Materials and Methods

### 2.1. Cells and Animals

4T1 breast cancer cells were purchased from the cell bank of Xiangya Medical College of the Central South University and cultured in RPMI-1640 (Invitrogen, USA) containing 10% fetal bovine serum (Tianhang biotechnology Co., Ltd., Zhejiang, China) at 37°C and 5% CO_2_ in a humidity saturated atmosphere.

32 female BABL/c mice (Changsha, Hunan, China, number: 2017-0029) were provided by the Experimental Animal Center of Silaike Co., Ltd. The mice were 4 weeks old, weighting 18 – 20 g. They were fed in a SPF environment that had regular ultraviolet radiation.

### 2.2. Preparation of the YHD Extract

All the herbs were obtained from the Liuyang Hospital of TCM. Herbs (46 g), including 30 g of Shudi, 3 g of Rougui, 6 g of Baijiezi, 2 g of Mahuang, 2 g of Jiangtan, and 3 g of Shenggancao, were blended into distilled water (about 5-fold the weight of the mixture) for 30 min and then heated at 100°C for 1 hour, after which the residue was boiled for 1 hour with distilled water. The decoction was mixed and Lujiaojiao (9 g) was melted into it. 2.86 g/ml of herb water extract [19] was obtained from the mixed decoction and filtered with a 0.2 *μ*m filter. The extract was stored at −20°C until used.

### 2.3. Model and Drug Use

The 4T1 breast cancer cells of the logarithmic growth were digested with Trypsin (HyClone, Thermo Scientific, USA) and adjusted to 5 × 10^7^/ml with a Phosphate Buffer Solution (PBS) (Senbeijia biotechnology Co., Ltd., Nanjing, China). Each mouse was inoculated with 0.2 ml cell suspension in the right armpit. A week after implantation, the 32 mice in which tumors had grown between 0.4 and 0.6 cm in diameter were grouped randomly into 4 groups (8 mice per group): a control group (NS 0.4 ml via gavage), a Cyclophosphamide (CTX) (Hengrui medicine Co., Ltd., Jiangsu, China) group (CTX 0.2 ml 100 mg/kg via intraperitoneal injection), a low-dose group (YHDL 0.4 ml 1.43 g/ml via gavage), and a high-dose group (YHDH 0.4 ml 2.86 g/ml via gavage). The mice were euthanized after 14 days of administration. Blood samples were taken by eyeball removal and the serum was separated using a centrifugal machine (Sorvall ST 40, Thermo Scientific, USA). The tumor tissues were weighted and the inhibition rate of the tumor was calculated using the following formula:

(1) $$\begin{array}{c} \text{Inhibition rate}=\frac{W\text{ control}-W\text{ treatment}}{W\text{ control}}\times 100\% \end{array}$$

W_control_ and W_treatment_ are the tumor weights of control and treatment group, respectively.

### 2.4. Flow Cytometry (FCM)

The number of MDSCs, NKT cells, and T cells subsets were detected using FCM. Tumor tissues were cut into small fragments, digested with 0.25% Trypsin for 30 min, and then filtered using 70 *μ*m cell strainers. This single-cell suspension was then centrifuged at 1500 rpm for 5 min. The supernatant was discarded and the cell concentration was adjusted to 5 ×10^8^/ml. The cell suspension was then washed using a mouse tumor-infiltrating lymphocyte separation medium (CW0049S; CWBIO, Jiangsu, China) and centrifuged at 1500 rpm for 15 min to obtain the lymphocytes. Lymphocytes with a concentration of 2 × 10^6^/ml were collected and washed with PBS, followed by staining with anti-CD11b FTIC (No. 557397; BD, USA), anti-LY6G (No. 553989; BD, USA) and LY6C PE (No. 553126; BD, USA) (Gr-1), anti-CD3e FTIC (No. 46003 280; eBioscience, USA), anti-CD49a PE (No. 130107632; BD, USA), anti-CD4 FTIC (No. 11004282; eBioscience, USA), anti-CD8a PE (No. 11008182; eBioscience, USA), and anti-CD3 PerCP-CY 5.5 (No. 46 003280; eBioscience, USA), along with the appropriate isotype controls. The cells were analyzed using a flow cytometry FACSCalibur (BD, USA).

### 2.5. Enzyme Linked Immunosorbent Assay (ELISA)

IFN-*γ*, TGF-*β*, and IL-6 levels in peripheral blood were quantitated using ELISA, according to the manufacturer's instructions: the IFN-*γ* ELISA kit (EK0375; R&D Systems, USA); the TGF-*β* ELISA kit (EK0515; R&D Systems, USA); and the IL-6 ELISA kit (EK0515; R&D Systems, USA).

### 2.6. Quantitative Real-Time PCR (RT-qPCR)

The expression of iNOS and ARG-1 was detected using a RT-qPCR assay. Two MDSC subsets, M-MDSCs and G-MDSCs, were isolated from the cell suspension which was obtained from the tumor tissue using an anti-CD11b antibody conjugated with biotin in combination with magnetic beads (Miltenyi, Germany) according to the manufacturer's instructions. The total RNA was extracted from isolated MDSCs and reverse transcribed to cDNA using a Power SYBR Green Cells-to-CT kit (A3500; Promega, USA). Quantitative PCR was applied using a SYBR green supermix (730003407; Bio-Rad, USA). The relative change in gene expression was analyzed using the 2^–ΔΔCt^ method using *β*-actin as the internal reference. The forward and reverse primer sequences were as follows: iNOS (forward TGAACCCCAAGAGTTTGACC, reverse TGCTGAAACATTTCCTG TGC), ARG-1 (forward GGTTGCCAAGCCTTATCGGA, reverse ACCTGCTCCACTGCC T TGCT), and *β*-actin (forward TGTGTC CGTCGTGGATCTGA, reverse CCTGCTTCACCAC CTTCTT GA).

### 2.7. Western Blot (WB) Analysis

The protein expression of STAT1, p-STAT1, STAT3, and p-STAT3 were detected using a WB assay. Total protein was extracted from the MDSCs by using a radio-immunoprecipitation assay buffer (CW2334S; CWBIO, Jiangsu, China) and determined by a BCA protein assay (CW0014S; CWBIO, Jiangsu, China). 50 *μ*g of protein was separated by 10% SDS-PAGE (CW0027S; CWBIO, Jiangsu, China) and then transferred into PVDF membranes. The membranes were blocked by 5% nonfat milk at room temperature for 1 hour, and then overnight at 4°C with the primary antibodies (SA00001-1; 1:2000; Proteintech Group, USA). The membranes were incubated at room temperature for 1 hour with secondary antibodies (SA00001-2; 1:2000; Proteintech Group, USA) the next day. The signal intensity was analyzed using the software Image J.

### 2.8. Statistics

All experimental data were analyzed with SPSS 18.0. Data are presented as the mean ± SEM. The significance of the differences between groups was analyzed using one-way analysis of variance (ANOVA) followed by the least significant difference test between each group.* p* < 0.05 was considered statistically significant.

## 3. Results

### 3.1. The Effect of YHD on Tumor Weight

**Figure 1:**
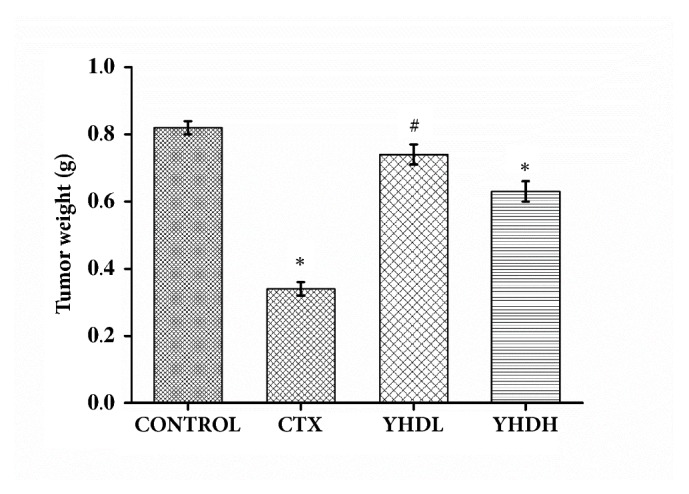
Tumor weight of the CTX group and different doses of the YHD groups. The bar chart shows the change in tumor weight over medication time. Data are expressed as the mean ± SEM of 8 mouse (*∗p* < 0.05 compared with the control group).

Compared with the control group, the tumor weight of the YHDH group and the CTX group was significantly reduced (*p* < 0.05) (Figure 1). The tumor-inhibition rates of the CTX, YHDL, and YHDH groups were 58.5%, 9.7%, and 23.2%, respectively.

### 3.2. The Effects of YHD on MDSCs, NKT Cell, and T Cell Subsets in the Tumor Microenvironment

**Figure 2:**
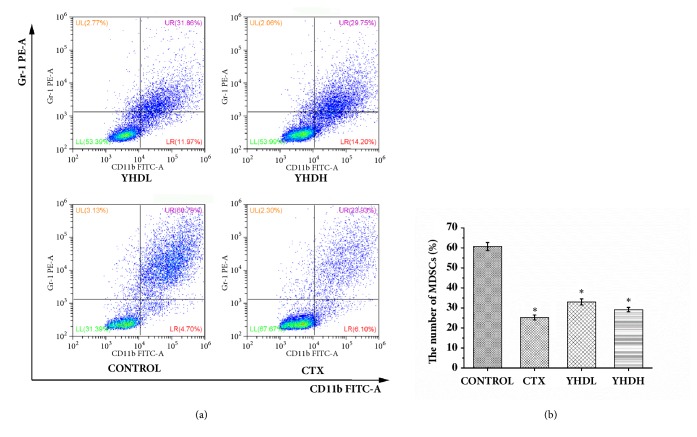
The number of MDSCs in the tumor microenvironment detected by flow cytometry. (a) The results of FCM. (b) The bar chart shows the inhibition effect of CTX and YHD on MDSCs in tumor tissue sections. Data are expressed as the mean ± SEM of 3 experiments (*∗p* < 0.05 compared with the control group).

The number of MDSCs in the tumor microenvironment of the YHD groups and the CTX group was significantly lower than the control group (*p*< 0.05) (Figure 2). Compared with the CTX group, the number of MDSCs in the YHD groups was not significantly different during the experiment (*p* > 0.05). The results show that both CTX and YHD could inhibit the proliferation of MDSCs.

**Figure 3:**
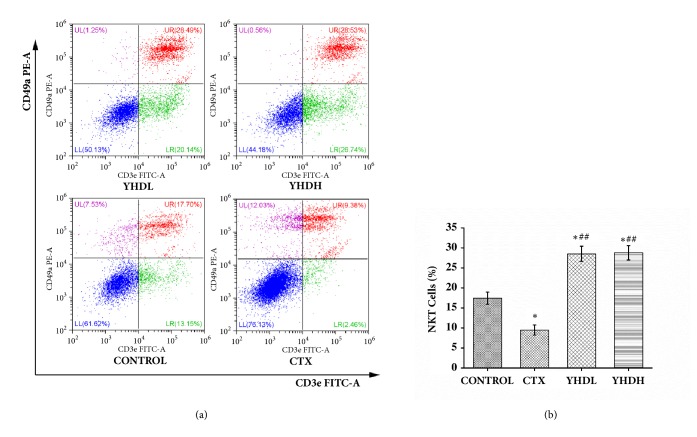
The number of NKT cells in tumor microenvironment detected by flow cytometry. (a) The results of FCM. (b) The bar chart indicated the opposite effect of CTX and YHD on NKT cells in the tumor tissue. Data were expressed as the mean ± SEM of 3 experiments (*∗p* < 0.05 compared with the control group; ^##^*p* < 0.01 compared with the CTX group).

**Figure 4:**
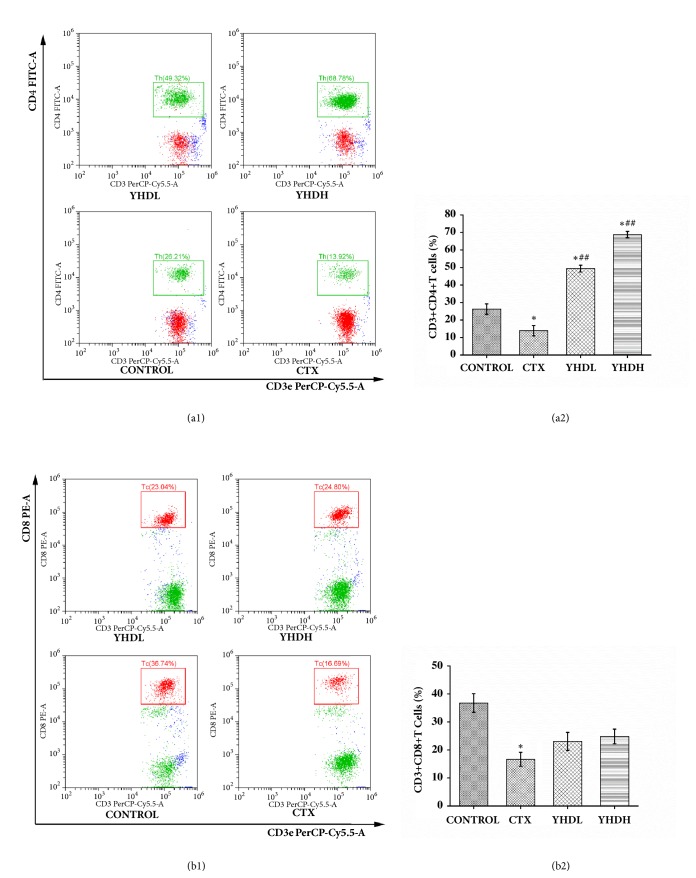
The number of T cells subsets in tumor microenvironment detected by flow cytometry. (a1) The results of FCM on the number of CD3^+^CD4^+^T cells. (a2) The number of CD3^+^CD4^+^T cells. The bar chart indicated the opposite effect of CTX and YHD on CD3^+^CD4^+^T cells in the tumor tissue. (b1) The results of FCM on the number of CD3^+^CD8^+^T cells. (b2) The number of CD3^+^CD8^+^T cells. The bar chart indicated inhibition effect of CTX on CD3^+^CD8^+^T cells. Data were expressed as the mean ± SEM of 3 experiments (*∗p* < 0.05 compared with the control group; ^##^*p* < 0.01 compared with the CTX group).

Compared with the control group, the number of NKT cells and CD4^+^T cells significantly increased in the YHDL group and the YHDH group (*p* < 0.05), while they declined in the CTX group (*p* < 0.05). Compared with the CTX group, the number of NKT cells and CD4^+^T cells significantly increased in the YHD groups (*p* < 0.01) (Figures 3 and 4(a)). Compared with the control group, the number of CD8^+^T cells declined in the CTX group (*p* < 0.05), while no significant difference was found in the YHD groups (Figure 4(b)). The data shows that YHD made a positive contribution to the immune system; on the contrary, CTX worked against it.

### 3.3. The Effect of YHD on the Expression of IFN-*γ*, TGF-*β*, and IL-6 in Peripheral Blood

**Figure 5:**
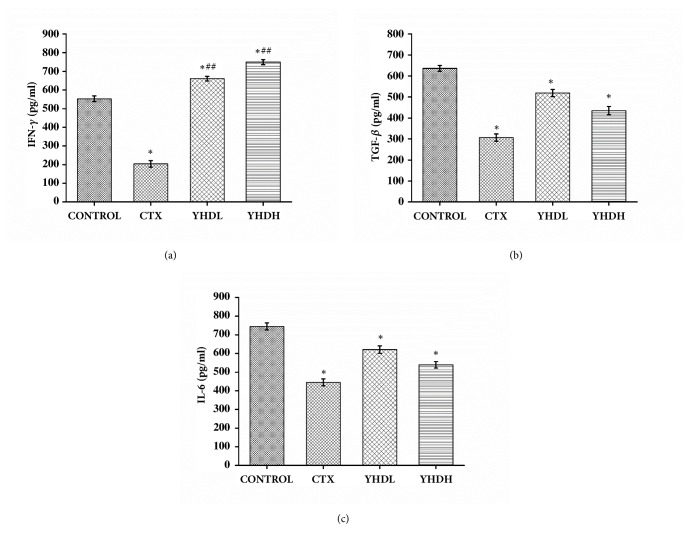
The expression of IFN-*γ*, TGF-*β*, and IL-6 in peripheral blood detected using ELISA. (a) The expression of IFN-*γ*. The bar chart shows the opposite effect of CTX and YHD on the expression of IFN-*γ* in peripheral blood. (b), (c) The bar charts show the inhibition effect of CTX and YHD on both of TGF-*β* and IL-6. Data are expressed as the mean ± SEM of 3 experiments (*∗p* < 0.05 compared with the control group, ^##^*p* < 0.01 compared with the CTX group).

In all treatment groups the expression of TGF-*β* and IL-6 in peripheral blood significantly decreased compared with the control group (*p* < 0.05). Compared with the CTX group, no significant difference was found in YHD groups on the level of TGF-*β* and IL-6 (*p* > 0.05) (Figures 5(b) and 5(c)). The results showed that both CTX and YHD could repress the expression of TGF-*β* and IL-6 on peripheral blood. Compared with the control group, the level of IFN-*γ* in the CTX group dramatically decreased (*p* < 0.01) and, on the contrary, increased in the YHD groups (*p* < 0.05), which indicated that the CTX and YHD regulate the expression of IFN-*γ* in a perverse way (Figure 5(a)).

### 3.4. The Effect of YHD on mRNA the Expression of iNOS and ARG-1

**Figure 6:**
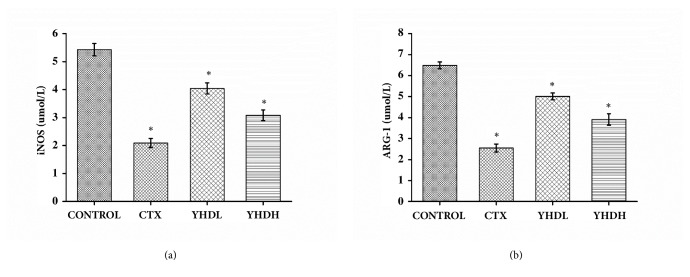
The expression of iNOS and ARG-1 in the tumor microenvironment detected by RT-PCR. (a) The expression of iNOS. (b) The expression of ARG-1. The bar chart shows that both CTX and YHD could suppress the expression of iNOS and ARG-1 in tumor tissue. Data are expressed as the mean ± SEM of 3 experiments (*∗p* < 0.05 compared with the control group).

In all treatment groups the mRNA expression of iNOS and ARG-1 in MDSCs decreased compared with the control group (*p* < 0.05). The results illustrated that, compared with YHD, CTX more strongly inhibited the expression of iNOS and ARG-1 (Figure 6), but no significant difference was found in YHD groups and CTX group on mRNA expression of iNOS and ARG-1(*p* >0.05).

### 3.5. The Effect of YHD on Proteins Expression of p-STAT1, STAT1, p-STAT3, and STAT3

**Figure 7:**
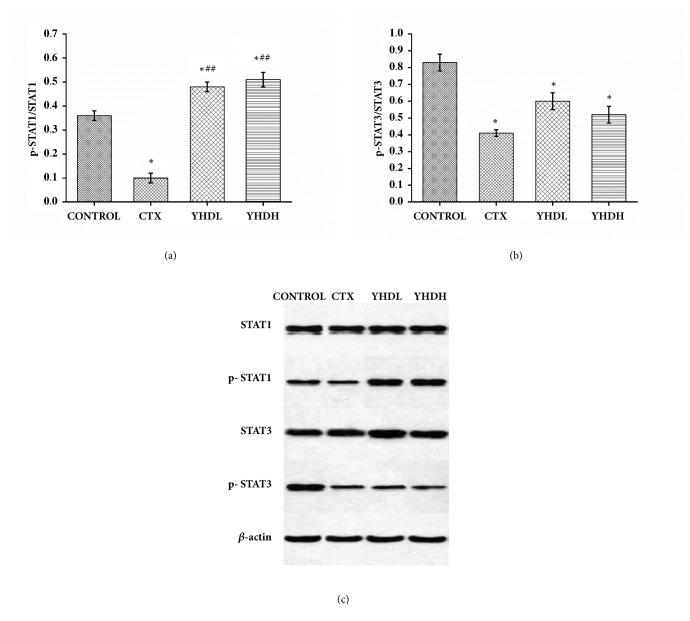
The expression of p-STAT1, STAT1, p-STAT3, and STAT3 in tumor tissue detected by western blot. (a) The expression of p-STAT1/STAT1. (b) The expression of p-STAT3/STAT3. (c) The protein expression of p-STAT1, STAT1, p-STAT3, and STAT3. Data are expressed as the mean ± SEM of 3 experiments (*∗p* < 0.05 compared with the control group; ^##^*p* < 0.01 compared with the CTX group).

Compared with the control group, in all treatment groups the protein expression of p-STAT3/STAT3 significantly decreased (*p* < 0.05). Compared with the control group, the level of p-STAT1/STAT1 declined in the CTX group and increased in the YHD groups (*p* < 0.05). Compared with the CTX group, the level of p-STAT1/STAT1 significantly increased (*p* < 0.01) (Figure 7).

## 4. Discussion

YHD has been widely used among Asians for the treatment and adjuvant treatment of breast cancer. Its advantages in cancer-preventive activity are in a multitarget, multibiological process and multipathway manner, which contributes to a positive effect but reduced toxicity and side effects. In this study, we treated the 4T1 mouse breast cancer model with YHD for 2 weeks; the results showed that YHD could inhibit the growth of 4T1 breast tumors.

MDSCs consist of myeloid progenitor cells, immature granulocytes, immature macrophages, and immature dendritic cells, which play an important role in tumor-associated immunosuppressive function. MDSCs mainly lead to tumor cell escape from immunosurveillance by inhibiting the function of T cells and NK cells. A large number of studies have demonstrated an increase in MDSCs in breast cancer [20], urothelial carcinoma [21], and glioblastoma [22]. Immune suppression by MDSCs involves multiple mechanisms. One of the crucial immunosuppressive mediators is ARG-1[23]. The activity of ARG-1 upregulation causes the decomposition of arginine, which leads to T cell cycle arrest in G_0_-G_1_ [24]; furthermore, T cell anergy is induced by the downregulation of T cell receptor (TCR) *ζ*-chain expression [25]. Besides ARG-1, another downstream product of MDSCs is iNOS, which also catabolizes arginine and then finally leads to T cell anergy. Other suppressive mechanisms of MDSCs are associated with producing immunosuppressive cytokines such as IL-10 and TGF-*β* [26], inducing T regulatory cells (Tregs) [27] and affecting NK cell function [28].

The antitumor effects of TCM herbs are widely known. The difference between TCM and chemotherapy is that TCM herbs not only directly kills tumor cells but also efficiently enhances the immune response. T lymphocytes, notably related to tumor adaptive immune cells, are mainly divided into two hypotypes according to their function and phenotypes: T helper cells (Th: CD3^+^CD4^+^ T cells) and T cytotoxic cells (Tc:CD3^+^CD8^+^ T cells). CD4^+^ T cells can meditate the bioactivity of other cells in the immune network by secreting cytokines, which play a key role in the initiation of immune responses and the strength of the immune system. CD8^+^ T cells release perforin and particle enzymes to kill tumor cells. A study has shown that the TCM formula Shugan Jianpi decoction had an inhibition function in MDSC proliferation and could enhance the inflammatory functions of NKT cells, which were associated with MDSC regulation [15]. Some ingredients of TCM herbs such as Astragaloside, extracted form Huangqi (Radix Astragali), could significantly increase IFN-*γ* secretion of T cells and promote T cell immune activity [16].

The present study found that YHD could decrease the number of MDSCs and downregulate the expression of iNOS and ARG-1 in the tumor microenvironment. Meanwhile, they also enhance the immune response by increasing the number of NKT cells and CD4^+^ T cells. Furthermore, the underlying mechanisms are discussed. The JAK/STAT signaling pathway is well-known for its key role in a wide variety of physiological and biological processes [29]. IFN-*γ* is a soluble cytokine which can enhance the antitumor immune response by activating the JAK/STAT1 pathway. On the other hand, the IL-6/JAK/STAT3 autocrine activation loop is a critical driver of cancer progression and metastasis in breast cancer [30, 31]. More importantly, activation of the JAK/STAT3 pathway is a necessary condition for the recruitment of MDSCs to tumor cells and exertion of their immunesuppression. Besides, TGF-*β* can also recruit MDSCs via activating the JAK/STAT3 pathway. Based on the results of this study, the expression of CD4^+^ T cells, NKT cells, IFN-*γ*, and p- STAT1 increased, while the expression of IL-6, TGF-*β*, iNOS, ARG-1, and p- STAT3 decreased, with YHD treatment.

## 5. Conclusions

This study explored the mechanisms of antitumor and immunomodulatory effects of the YHD on breast cancer. On one hand, YHD inhibited the growth of 4T1 breast tumors via decreasing the number of MDSCs in the tumor microenvironment and through specific mechanisms associated with the downregulation of the expression of iNOS, ARG-1, IL-6, TGF-*β*, and p-STAT3. On the other hand, YHD enhanced the antitumor immune response via upregulation of NKT cells, CD4^+^T cells, IFN-*γ*, and p-STAT1. Therefore, this study provides new evidence of the effects of YHD on the treatment of breast cancer.

## Data Availability

The data used to support the findings of this study are available from the corresponding author upon request.
